# Phytochemical Characterization and Efficacy of *Artemisia judaica* Extract Loaded Chitosan Nanoparticles as Inhibitors of Cancer Proliferation and Microbial Growth

**DOI:** 10.3390/polym15020391

**Published:** 2023-01-11

**Authors:** Husam Qanash, Abdulrahman S. Bazaid, Abdu Aldarhami, Bandar Alharbi, Majed N. Almashjary, Mohannad S. Hazzazi, Hashim R. Felemban, Tarek M. Abdelghany

**Affiliations:** 1 Department of Medical Laboratory Science, College of Applied Medical Sciences, University of Ha’il, Hail 55476, Saudi Arabia; ar.bazaid@uoh.edu.sa (A.S.B.); b.alharbi@uoh.edu.sa (B.A.); 2 Department of Medical Microbiology, Qunfudah Faculty of Medicine, Umm Al-Qura University, Al-Qunfudah 21961, Saudi Arabia; ahdarhami@uqu.edu.sa; 3 Department of Medical Laboratory Sciences, Faculty of Applied Medical Sciences, King Abdulaziz University, Jeddah 22254, Saudi Arabia; malmashjary@kau.edu.sa (M.N.A.); mshazzazi@kau.edu.sa (M.S.H.); hrfelemban@kau.edu.sa (H.R.F.); 4 Hematology Research Unit, King Fahd Medical Research Center, King Abdulaziz University, Jeddah 22254, Saudi Arabia; 5 Special Infectious Agents Unit-BSL3, King Fahd Medical Research Center, King Abdulaziz University, Jeddah 21362, Saudi Arabia; 6 Botany and Microbiology Department, Faculty of Science, Al-Azhar University, Cairo 71524, Egypt

**Keywords:** Artemisia, chitosan nanoparticles, anticancer, antimicrobial, bio-efficacy

## Abstract

Despite the advanced development in the field of drug discovery and design, fighting infectious and non-infectious diseases remains a major worldwide heath challenge due to the limited activity of currently used drugs. Nevertheless, in recent years, the approach of designing nanoparticles for therapeutic applications has gained more interest and promise for future use. Thus, the current study is focused on the evaluation of *A. judaica* extract and chitosan nanoparticles loaded extract (CNPsLE) for potential antimicrobial and anticancer activities. The HPLC analysis of the extract has shown the presence of various phenolic and flavonoid compounds, including kaempferol (3916.34 µg/mL), apigenin (3794.32 µg/mL), chlorogenic acid (1089.58 µg/mL), quercetin (714.97 µg/mL), vanillin (691.55 µg/mL), naringenin (202.14 µg/mL), and rutin (55.64 µg/mL). The extract alone showed higher MIC values against *B. subtilis*, *E. coli*, *S. aureus*, *K. pneumonia*, and *C. albicans* (62.5, 15.65, 15.62, 31.25, and 31.25 µg/mL, respectively), whereas lower MIC values were observed when the extract was combined with CNPsLE (0.97, 1.95, 3.9, 4.1, and 15.62 µg/mL, respectively). The extract exhibited low cytotoxicity against normal Vero cells with IC_50_ 173.74 µg/mL in comparison with the cytotoxicity of the CNPsLE (IC_50_, 73.89 µg/mL). However, CNPsLE showed more selective toxicity against the human prostate cancer cell line (PC3) with IC_50_ of 20.8 µg/mL than the extract alone with 76.09 µg/mL. In the docking experiments, kaempferol and apigenin were revealed to be suitable inhibitors for prostate cancer (2Q7L). Overall, the obtained data highlighted the promising potential therapeutic use of CNPsLE as an anticancer and antimicrobial agent.

## 1. Introduction

Plants are a rich source for diverse chemical compounds with various therapeutic properties that can be used alone or in combination with other medicines to treat infectious and non- infectious diseases [1,2,3]. Although numerous plant-derived products have been discovered and clinically used, this source still offers high probability for the discovery of new and promising therapeutic compounds for treating human illness. *Artemisia* is one of the largest genera within the Asteraceae family and the leading genus within the species, comprising between 200 and greater than 500 taxa at the species or sub-species level. For instance, *mugwort*, *wormwood*, and *sagebrush* are well-known species of *Artemisia*. Multiple species of *Artemisia* have gained great interest in various scientific fields in the search for promising heath and economical outcomes due to its reported nutritional and biological properties. In addition, numerous compounds are produced by species of this genera, including flavonoids, alkaloids, phenols, quinines, and terpenoids, exhibiting various biological activities [4]. This involves anticancer, antidepressant, anxiolytic, antidiabetic, antitubercular, hepatoprotective, gastroprotective, antihypertensive, antiepileptic, insecticidal, antiemetic, antihyperlipidemic, antiparasitic, and antiviral activities [5].

*Artemisia judaica* L. is one of the important species of *Artemisia* commonly grown in the Mediterranean area, including Saudi Arabia, Egypt, Algeria, Libya, and Jordan [6]. *A. judaica* was reported to exhibit great potential therapeutic use for gastrointestinal disorders, inflammatory disorders, sexual dysfunction, heart diseases, hyperglycemia, cancers, arthritis, oxidative stress, and wound healing [7,8]. In addition, *in vitro* investigations have reported anticancer and antioxidant activities of *A. judaica* extracts. Furthermore, antimicrobial activity of *A. judaica* oil was observed against *Candida albicans*, *Cryptococcus neoformans* [9], *Bacillus cereus*, and *Aspergillus niger* [8]. Moreover, both aqueous or alcoholic extracts of *A. judaica* have decreased the level of blood glucose in diabetic rats [10], showing higher antioxidant activity compared to ascorbic acid [11].

Recently, the field of nanotechnology has gained more interest regarding nanoparticle design and/or its incorporation with plant extract to enhance its biological activities [12]. The combination of certain natural extracts, such as oregano, savory, marjoram [13], sage [14], *Leucas aspera* [15], *Pterocarpus marsupium* [16], and *aloe vera* gel [17], with chitosan nanoparticles (CNPs) was performed and shown to increase the activity of the extract. The unique characteristics of CNPs, including surface their area, the existence of active functional groups, limited toxicity, great permeability into bacterial cell membranes, and cost effectiveness, have prioritized their potential therapeutic applications in comparison with bulk chitosan [18]. The antifungal activity of a *Byrsonima crassifolia* essential oils loaded with CNPs was reported against *Colletotrichum gloeosporioides* and *Alternaria* species [19]. In addition, the anticancer activity of essential oils from a species of *Artemisia* incorporated with CNPs against breast cancer lines was documented, while unloaded essential oils showed less anti-proliferative activity [20]. In addition, although *Artemisia* scoparia extract displayed limited toxicity towards hepatocarcinoma cell lines, its activity was notably improved when combined with chitosan-myristate nanogel [21]. Moreover, a previous in vitro study claimed the significant enhancement by CNPs for the anticancer activity of *Zataria multiflora* essential oil against breast and melanoma cells [22]. Although *Artemisia* species are well reported as being a rich source of compounds with promising nutritional and therapeutic properties, the enhancing, expanding, and stabilizing of desired biological activities is urgently required to overcome the resistance, ineffectiveness, and instability of currently and clinically used antimicrobial or anticancer compounds. Thus, the current study investigates the chemical composition of tested *Artemisia* species, evaluating the antimicrobial and anticancer activities of the extract alone, as well as in combination with chitosan nanoparticles.

## 2. Materials and Methods

### 2.1. Source of Chitosan Nanoparticles

High quality chitosan nanoparticles (CNPs) powder was purchased from Primex, Siglufjordur, Iceland. Analytical grade solvents, buffers, and reagents used were obtained from Sigma-Aldrich (St. Louis, MS, USA), while Dimethyl Sulfoxide (DMSO) was purchased from Riedel-de Haën -Honeywell Research Chemicals (Seelze, Germany).

### 2.2. Plant Collection and Extraction

The aerial parts of *Artemisia judaica* were collected (May 2022) and dried, then ground into fine powder using a manual mortar. In a Soxhlet extractor (Fisher Scientific, Leicestershire, UK), about 5 g of powder was extracted using 250 mL of methanol over a one-day period. The extraction was repeated for three rounds, followed by evaporation via a rotary evaporator (Henan Lanphan Industrial Co., Ltd., China), yielding 0.6 mg g^−1^ as a crude extract. The extract was subjected to high-performance liquid chromatography (HPLC) analysis and the evaluation of its biological activities, including antibacterial, antifungal, and anticancer activities was determined.

### 2.3. Loading of A. judaica into Chitosan Nanoparticles

The loaded nanoparticles were purified by centrifugation, and the diameter of chitosan nanoparticles (CNPs) was then assessed. Briefly, CNPs solution was prepared through dissolving CNPs in an aqueous solution of acetic acid (1%, *v*/*v*), followed by the adding of CNPs to get 2% (*w*/*v*) as a final concentration. To the prepared solution, glycerol was added at a concentration of 2% (*w*/*v*) as a plasticizer. Then, Tween 20 (0.05%, *v*/*v*) was added to enhance the adhesion and wettability properties. To prepare extract incorporated with CNPs coating solution, the extract was incorporated into CNPs solution (stirred for 25 min) to obtain the final concentration (10%) of extract concentrate [17].

### 2.4. HPLC Analysis

Following the extraction, 5 μL of the *A. judaica* extract was injected into the HPLC machine (Agilent 1260 series, Agilent Technologies, Santa Clara, CA, USA). The Eclipse C18 column was used as follows: 4.6 mm × 250 mm i.d., and 5 μm that was maintained at 40 °C. Two buffers were used as the mobile phase—Buffer A (mili Q water + 0.05% trifluoroacetic acid) and Buffer B (acetonitrile + 0.05% trifluoroacetic acid—at a flow rate of 0.9 mL/minute. The mobile phase was programmed in a linear gradient for 20 min run as follows: 0 min (82% Buffer A); 0–5 min (80% Buffer A); 5–8 min (60% Buffer A); 8–12 min (60% Buffer A); 12–15 min (82% Buffer A); 15–16 min (82% Buffer A), and 16–20 (82% Buffer A). One wavelength at 280 nm was used by the ultraviolet (UV) detector (Icon Scientific Inc., North Potomac, M.D., USA) for the detection of phenolic and flavonoids contents. The phenolic and flavonoids contents of the extract were estimated qualitatively and quantitatively according to the methods of Abdelghany et al. [1], in comparison with the injected/used standards of phenolic flavonoid compounds.

### 2.5. Agar Well Diffusion Assay

Modified Mueller–Hinton agar and potato dextrose agar media were used for the growth of the tested bacteria and fungi, respectively. The agar well diffusion method was utilized to estimate the antimicrobial activity of the extract alone and when combined with CNPs. Bacterial isolates were collected from Ain Shams University Hospital, Egypt, while the mycology laboratory at Assiut University, Egypt, provided the research team with requested fungal isolates. The appropriate agar plate was individually inoculated with the tested microbe, including *Staphylococcus aureus* [ATCC 6538], *Bacillus subtilis* [ATCC 6633], *Escherichia coli* [ATCC 8739], *Klebsiella pneumonia*, *Aspergillus niger*, and *Candida albicans* [ATCC 10231], using sterile swabs. Next, a well (6 mm diameter) was formed using a sterile cork-borer, which was then filled with 100 µL of the tested sample. Then, agar plates inoculated with bacteria were incubated at 37 °C for 24 h, whereas about 72 h of incubation at 30 °C were used for fungal growth. The inhibitory zones around the wells were measured in millimeters (mm). Inhibitory zones caused gentamycin and clotrimazole against tested bacterial, and fungal isolates were used as positive controls, respectively [23].

### 2.6. Minimal Inhibitory Concentration (MIC)

The micro-dilution broth technique was applied to determine the minimal inhibitory concentration (MIC) (the lowest concentration of the sample that inhibits the growth of the tested microorganisms). A stock concentration was prepared to produce the multiple dilutions (0.98 to 1000 µg/mL) of the extract. A constant volume (200 µL) of each dilution was added to the designated well/s of a sterile 96-wells plate containing broth medium. The 0.5 McFarland suspension of each tested microorganism was prepared in 0.85% sterile sodium chloride (normal saline). About 2 µL of the prepared microbial suspension (containing 2 × 10^8^ of the bacterial colony forming units (CFU/mL, 2 × 10^6^ CFU/mL for yeasts and 4 × 10^6^ CFU/mL for filamentous fungi) was transferred to the designated well(s). Subsequently, the plates were incubated at the appropriate growth condition for bacteria and fungi, as mentioned above. Finally, MIC values were determined based on a visual observation of the microbial growth.

### 2.7. Cell Viability Assay

Prostate cancer (PC3) cells and normal Vero cells were obtained from Nawah Scientific (Inc., Cairo, Egypt), and about 1 × 10^5^ cells/mL (100 µL/ well) were inoculated in the 96-well tissue culture plate, then incubated at 37 °C, 5% CO_2_, for 24 h to develop a whole monolayer sheet. After that, the growth medium was decanted from the culture plate when the confluent sheet of the tested cells was formed. Then, the cell monolayer was washed using Roswell Park Memorial Institute (RPMI) medium with 2% serum (maintenance medium), which was amended with different dilutions (0.1 mL for each well) of the tested extract. The wells without any dilution but containing maintenance medium were used as controls. Plates were incubated at 37 °C, 5% CO_2_, overnight to investigate any possible cytopathic effects of treated cells. A solution of 3-(4,5-dimethylthiazol-2-yl)-2,5-diphenyltetrazolium bromide) (MTT) was prepared using phosphate buffered saline (PBS) as a solvent (5 mg/mL) [24]; then 20 µL of the prepared solution was added into each well, followed by well-mixing of prepared solution with the used medium through a shaking table at 150 rpm for 5 min [25]. The well plates were incubated at 37 °C for 4 h under 5% CO_2_ to initiate/enhance the metabolizing of MTT. Then, the metabolic product of MTT was resuspended in 200 µL of DMSO and left for 5 min on the shaking table at 150 rpm to enhance the mixture of the metabolic product with the solvent. At 560 nm and a subtracted background at 620 nm, the optical density was read to determine the viability of the treated cells [2,26]. Cell cytopathic effects were monitored using an inverted microscope.

### 2.8. Molecular Docking

The Molecular Operating Environment (MOE) is a set of software applications for the computer-aided design of the main detected kaempferol and apigenin flavonoids as a biologically active compounds in the *A. judaica* extract against the PC3 cell line. It enables the drawing of molecules and their minimization for the best possible conformations. The Androgen Receptor Prostate Cancer (2Q7L) was downloaded from the protein data bank (http://www.rcsb.org/pdb, assessed on 5 September 2022) [27]. The protein molecule performed 3D protonation after the water molecules were removed. To optimize the geometry of 2Q7L, the enzyme was shown using the MMFF94x force field implemented in the MOE software. The main chain was kept firm, while the side chains remained flexible. This approximation allowed the side chains of the proteins to find the position in which the interactions are most favorable. We then recorded the best score, that is, the one with the lowest energy corresponding to the best interactions between the ligands and the active site of the enzyme. The next indexes were used in the docking study: S = final score, which is the score of the last stage that was not set to none; rmsd = root mean square deviation of the pose, in Å, from the original ligand. This field occurred if the site description was the same as the description of the ligand; rmsd_refine = root mean square deviation from the earlier refinement and the later refinement of the pose; E_conf = energy of the conformer. If there was a refinement stage, this was the energy calculated at the end of the refinement. Note that for force field refinement, by default, this energy was calculated with the solvation option set to Born; E_place = score from the placement stage; E_score 1 and E_score 2 = scores from rescoring stages 1 and 2; E_refine = score from the refinement step, calculated to be the totality of the van der Waals electrostatic plus the solvation energies, according to the generalized Born solvation model (GB/VI).

### 2.9. Statistical Analysis

All measurements were performed in triplicate, and the results were calculated as mean values plus standard deviation (±SD).

## 3. Results and Discussion

### 3.1. Phenolic and Flavonoid Analysis

**Figure 1:**
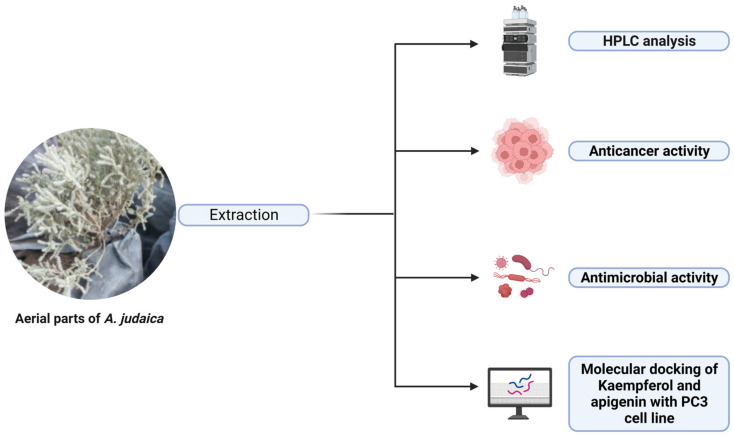
Image showing aerial parts (above the soil) of *Artemisia judaica* (plant), which underwent successful extraction methods and multiple relevant assays to determine the phytochemical, antimicrobial, and antitumor activities of the extracted product. This figure was created with BioRender.com.

**Figure 2:**
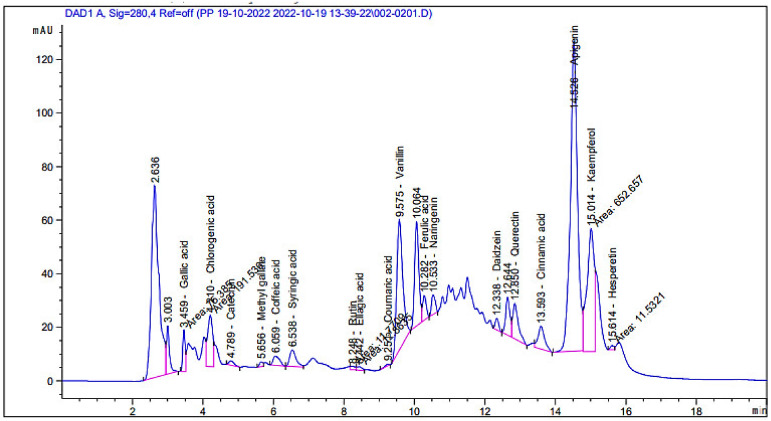
High-performance liquid chromatography chromatogram of polyphenolic and flavonoid compounds detected from *Artemisia judaica* extract.

**Figure 3:**
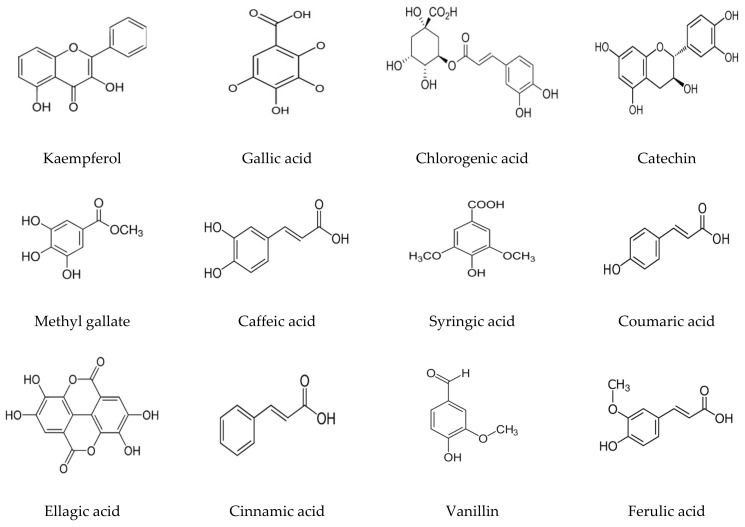

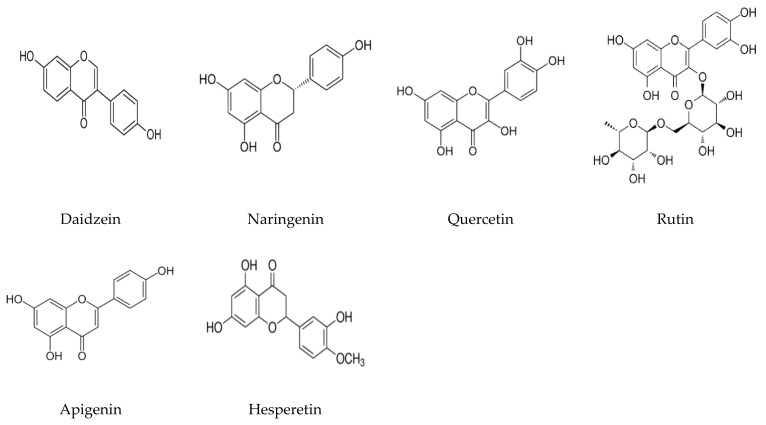
Chemical formulas of compounds detected in *Artemisia judaica* extract by high-performance liquid chromatography analysis.

Although the phytoconstituents of *A. judaica* were previously detected in several studies [28,29], they may differ based on growing conditions, climatic conditions, cultivar soil, the age of plant, and the flowering state; thus, the analysis of the plant extract was performed in this study. HPLC analysis for the *A. judaica* extract (aerial parts) revealed the presence of 18 known compounds (Figure 1), with their exact concentrations (Table 1 and Figure 2) and chemical formulas (Figure 3), but another 4 unknown compounds were also detected. Among the 18 known compounds, kaempferol, apigenin, and chlorogenic acid were revealed with the highest concentrations (3916.34, 3794.32, and 1089.58 µg/mL, respectively), while the lowest concentrations were noted for coumaric acid (5.46 µg/mL) and methyl gallate (27.68 µg/mL). The phytochemical profiles of the extract indicated that the aerial parts of *A. judaica* were very rich in phenolic and flavonoid compounds. Chlorogenic acid, luteolin, apigenin, rutin, and other compounds were reported in the extract of Artemisia gmelinii [4]. The identified compounds for *A. judaica* were reported, with various biological activities, i.e., kaempferol, which is a flavonoid compound, showed success toward the treatment of inflammatory responses, liver injuries, diabetes, malignancy, and obesity [30]. In addition, a recent study has claimed the promising potential therapeutic use of kaempferol, alone or in combination with colistin, to treat infections caused by drug resistant bacteria [31].

Apigenin is another compound identified with high concentrations in the A. *judaica* extract that was previously reported to exhibit anticancer, antioxidant, anti-inflammatory, and antimicrobial activities [32,33]. Similarly, multiple biological activities were linked with chlorogenic acid, including antibacterial activity towards *Yersinia enterocolitica* [34]. Other detected compounds in the current investigation, such as quercetin (714.97 µg/mL), naringenin (202.14 µg/mL), and rutin (55.64 µg/mL), were reported to possess great future possibilities for use as therapeutic agents for various illness. For instance, quercetin was shown to have antioxidant, anti-inflammatory, antidiabetic, anti-Alzheimer’s, and anticancer activities [35] [36]. Moreover, in vitro and in vivo studies of the naringenin compound have claimed its strong association with the suppression of cancer progression [37]. Furthermore, a recent report also proved multiple activities of rutin toward inflammation, oxidation, and thrombosis [38]. Although the above-mentioned activities noted for compounds within the current extract are not comprehensive, they should be adequate to state that *A. judaica*, especially its aerial parts, is very rich source for diverse compounds with various therapeutic properties.

### 3.2. Antimicrobial Activity

**Figure 4:**
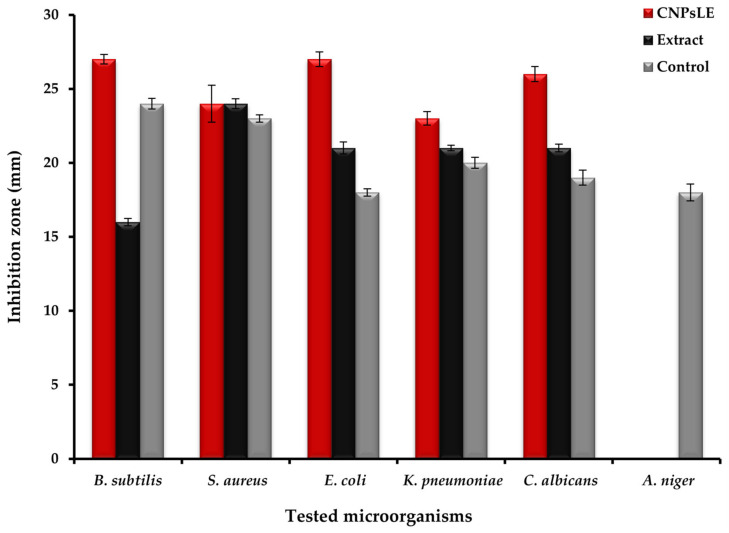
Antimicrobial activity of *Artemisia judaica* extract, chitosan nanoparticles loaded extract (CNPsLE), and chitosan towards multiple pathogenic bacterial and fungal species.

**Figure 5:**
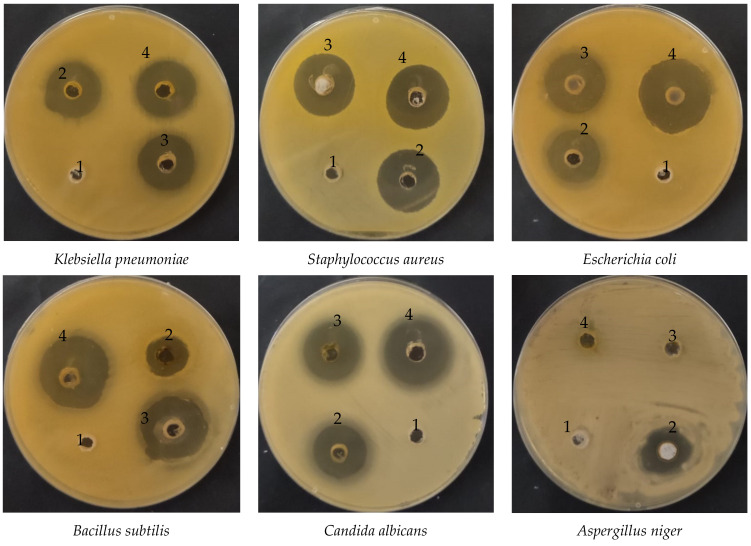
Well-diffusion assays to assess the [1] activity of chitosan, [2] positive controls (antibiotic/antifungal), [3] *Artemisia judaica* extract, and [4] chitosan nanoparticles (CNPs) loaded extract against pathogenic bacterial and fungal isolates.

Recent reports have claimed that CNPs possess a great promise for use in different medical applications through various technologies/approaches, including nanomedicine and biomedical engineering, for either enhancing their effectiveness or novel discovery [39,40]. CNPs loaded with *Artemisia judaica* extract (CNPsLE) were more effective than the extracts alone against all tested microorganisms, except *Staphylococcus aureus* (Figure 4 and Figure 5); this might be due to the differences in the cell wall compositions, virulence factors, or specific resistance mechanisms.

*Judaica* essential oils showed potent activity against the growth of *Aspergillus fumigatus*, *Geotrichum candidum*, *Syncephalastrum racemosum*, *Candida albicans*, *Escherichia coli, Bacillus subtilis*, and *Streptococcus pneumonia*, but failed to exhibit any activity towards the tested strain of *Pseudomonas aeruginosa* [41]. Nevertheless, another recent study [8], reported the high sensitivity of *Aspergillus niger*, *Candida albicans*, *Salmonella typhimurium*, and *Bacillus cereus* towards *A. judaica* extract, whereas *E. coli*, *Klebsiella pneumonia*, and *Shigella flexneri* were observed with limited susceptibility to the same extract [6]. This variation in the overall potency of the extract might be attributed to the solvent used in the extraction. For example, it was reported that no antifungal potential was observed on the tested fungi, with the exception of *C. albicans*. Moreover, ethyl acetate extract did not exhibit the growth of *A. fumigatus* and *Mucor* spp.; the growth of *C. albicans* and *A. fumigatus*, however, was detected by methyl alcohol extract. These outcomes are consistent with the current results. CNPsLE exhibited excellent MIC values against *B. subtilis* (0.97 µg/mL) *E. coli* (1.95 µg/mL), *S. aureus* (3.9 µg/mL), *K. pneumonia* (4.1 µg/mL), and *C. albicans* (15.62 µg/mL), but the same microbes showed higher MIC values—*B. subtilis* (62.5 µg/mL), *E. coli* (15.65 µg/mL), *S. aureus* (15.62 µg/mL), *K. pneumonia* (31.25 µg/mL), and *C. albicans* (31.25 µg/mL)—when treated with the extract only (Table 2). Extract from *Artemisia judaica*, without combination with any nanoparticle, was reported, with MIC between 2.5 and 5 mg/mL against *Candida* species, while species of Bacillus showed MIC between 1.25 and 5 mg/mL [4]. Currently, both the extract alone and the CNPsLE complex failed to inhibit the growth of A. niger (Figure 4 and Figure 5). This is in agreement with previous studies, in which either complete resistance (*Aspergillus niger* and *Alternaria alternate*) or limited sensitivity (*Fusarium solani*) of tested fungi towards *A. judaica* extract was noted [42]. In addition, the enhanced efficacy of the current extract, when combined with CNPs, towards the tested microbes was also observed for mint extract loaded with CNPs against the examined pathogenic fungi [43]. Moreover, *Torreya grandis* essential oil (EO) loaded on the CNPs (particle diameter, 349.6 nm) offered stronger antibacterial activity compared to the non-loaded EO [44]. Furthermore, *Achillea millefolium* extract loaded on the CNPs showed a three-fold activity against *B. subtilis* and *P. aeruginosa* compared to the extract alone [45]. Recently, CNPs incorporated with *aloe vera* gel demonstrated stronger activity against *Helicobacter pylori* [17]. This might be due to the small size and strong surface charge of the CNPs, which facilitates the easy and effective interaction between the microbial cell wall and the CNPs loaded with the active compound/s [46,47] Therefore, the use of the CNPs as carriers for plant extracts and other therapeutic agents would hold great promise for enhancing the activity of active agents or leading to the discovery of novel active compound(s), only when loaded into the CNPs.

### 3.3. Anticancer Activity

To measure the chitosan’s toxicity, PC3 and Vero cells were treated with chitosan alone. A negligible cytotoxicity of chitosan against the PC3 cell line was observed, in which the viability of 98.35% and 88.47% was reported at 10 µg/mL and 1000 µg/mL, respectively. However, no cytotoxicity against chitosan-treated Vero cells was recorded as showing a viability of 100% at 10 µg/mL, but the viability was then decreased to 96.22% at 1000 µg/mL. The cytotoxicity of the *A. judaica* extract and the CNPs loaded extract was then evaluated against the cancer and normal cell line simultaneously to determine the possible selective toxicity to tumor cells, as well as to assess the suitability of these compounds for potential use in human medicine. The extract alone showed less activity against normal Vero cells than the extract loaded on the CNPs, particularly at concentrations ≤ 125 µg/mL (Table 3). The growth of Vero cells was reduced by 0.25, 1.15, and 28.97% or 6.11, 54.37, and 86.90% when treated by 31.25, 62.5, and 125 µg/mL of the extract alone or the extract loaded on the CNPs, respectively. Namely, the IC_50_ of the extract alone was higher (173.74 µg/mL) than that calculated for the extract loaded on the CNPs (73.89 µg/mL) against normal Vero cells.

Anticancer assays demonstrated a higher efficacy of the extract loaded on the CNPs, especially at concentrations ≤ 125 µg/mL, compared to the extract alone against PC3 cells (Table 4). A promising anticancer activity (toxicity of 73.80%) of the extract loaded on the CNPs compared with the anticancer activity (toxicity of 14.80%) of the extract only at the same concentration (31.25 µg/mL) (Table 4) was noted; therefore, the IC_50_ was calculated as 20.8 and 76.09 µg/mL for the extract loaded on the CNPs and extract alone, respectively. *Artemisia quettensis* extract was reported to exhibit anticancer activity against the human colorectal adenocarcinoma cell line (HT29), with IC_50_ around 31.54 µg/mL at 24 h of exposure [48]. Regarding the enhanced anticancer activity of the current extract when combined with the CNPs, multiple previous similar studies have documented this phenomenon for the compound of natural source (extract) formulated with CNPs compared to the bulk extract alone [49]. For instance, CNPs doped with the sodium tripolyphosphate enhanced the anti-proliferation activity toward the human ovarian cancer cell line (PA-1), while absent cytotoxicity was noted towards the normal mouse embryo cell lines (3T3) [50]. The anticancer activity in the current study of the tested compounds was evaluated using the positive control (Adriamycin), yielding IC_50_, 58.07 µg/mL against PC3.

**Figure 6:**
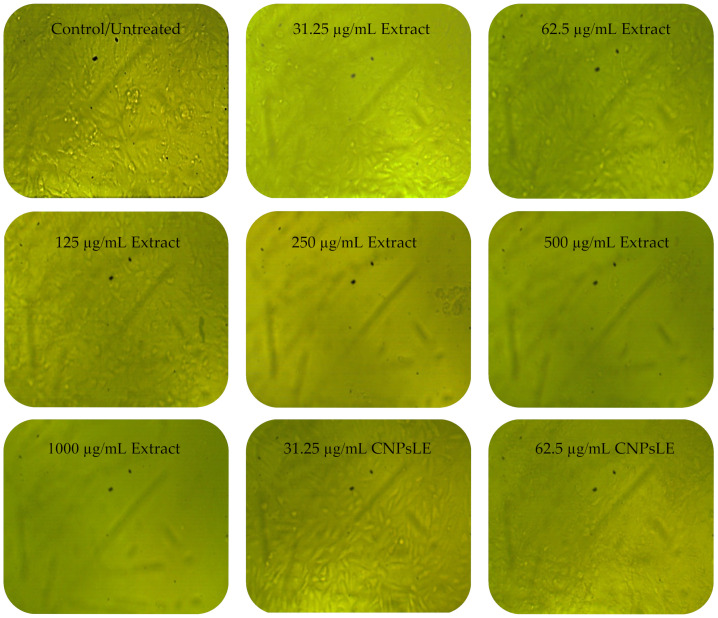

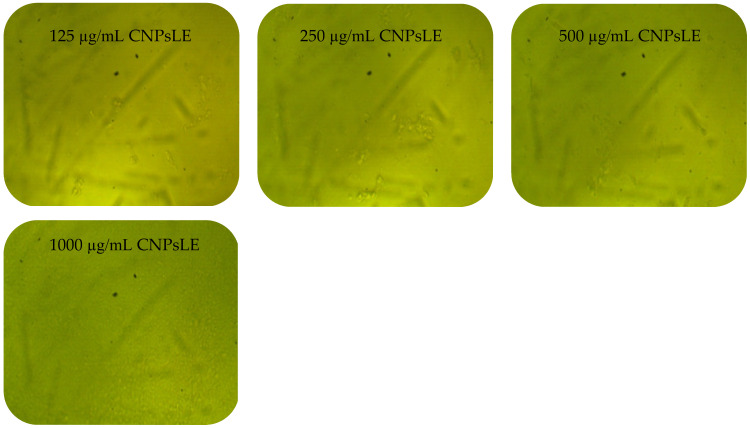
Images showing the cytopathic effect (CEP) of Vero cells treated with *Artemisia judaica* extract and *Artemisia judaica* extract loaded into chitosan nanoparticles (CNPsLE) at multiple concentrations.

**Figure 7:**
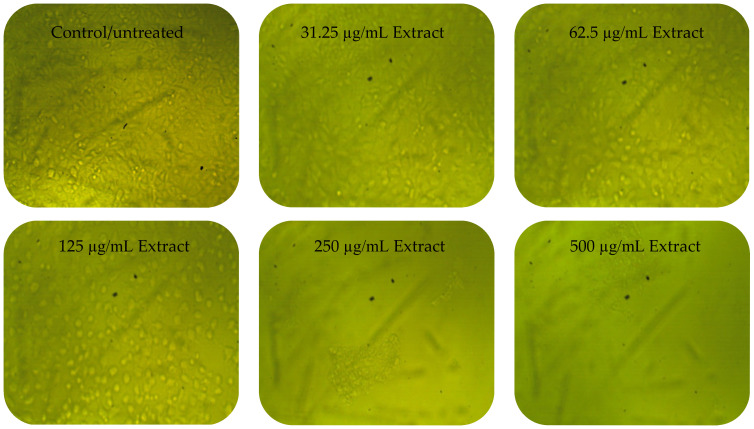

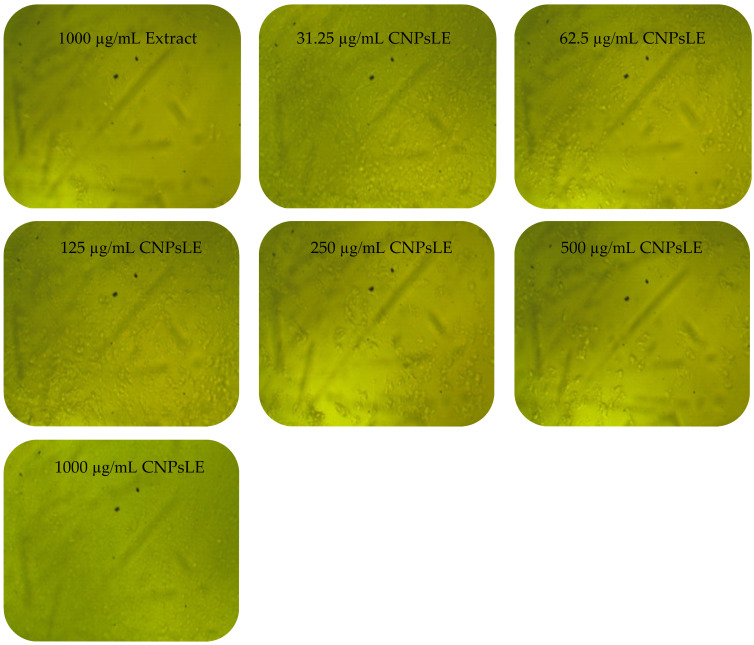
Images showing the cytopathic effects (CEP) of prostate cancer (PC3) cells treated with *Artemisia judaica* extract and *Artemisia judaica* extract loaded into chitosan nanoparticles (CNPsLE) at multiple concentrations.

Cytopathic effects (CPEs) were not observed on normal Vero cells treated with the *A. judaica* extract ≤ 62.5 µg/mL, while moderate CPEs were documented at 125 µg/mL (Figure 6). In addition, CPEs and the reduction of viable cells, or the complete absence and destruction of cells, was noted at 250–500 and 1000 µg/mL of the extract, respectively. The CNPsLE caused more morphological changes in the Vero cells at high concentrations 125–1000 µg/mL (Figure 6). Limited CPEs were observed on PC3 at lower concentrations of the *A. judaica* extract, but moderate morphological changes appeared, particularly at 250 to 1000 µg/mL (Figure 7). On the other hand, PC3 exposed to CNPsLE showed alteration in the shape, cell shrinkage, and membrane blebs at various tested concentrations. Apoptosis, the reduction of viable cells, and cytoplasmic condensation of treated PC3 cell with CNPsLE was observed at higher concentrations between 250 to 1000 µg/mL (Figure 7).

Although the *A. judaica* extract showed a lower affect towards all tested healthy and cancerous cells compared to the extract loaded on CNPs, both tested agents (*A. judaica* extract and CNPsLE) generally displayed more potent activity towards tested cancer cells (PC3) than the normal Vero cells. This potentially would indicate a possible selective toxicity of compounds detected from the *A. judaica* extract, although enhanced activity was observed when detected compounds from the *A. judaica* extract were loaded on the CNPs. Thus, detected compounds from the *A. judaica* extract, alone or combined with CNPs, show great promise for future use to treat prostate cancer, as their IC_50_ values are higher towards heathy cells (limited toxicity) compared to cancer cells.

### 3.4. Molecular Docking of Kaempferol and Apigenin with Prostate Cancer (2Q7L)

**Figure 8:**
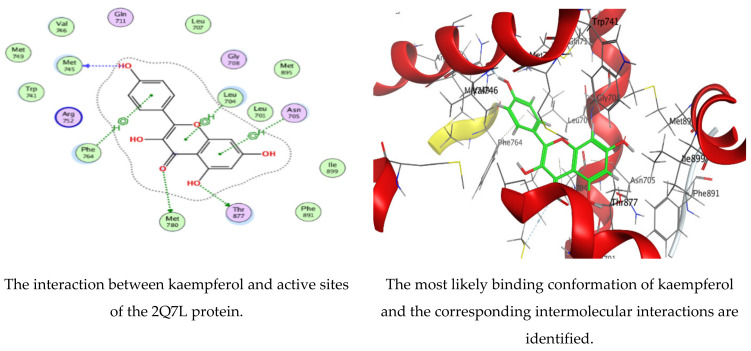

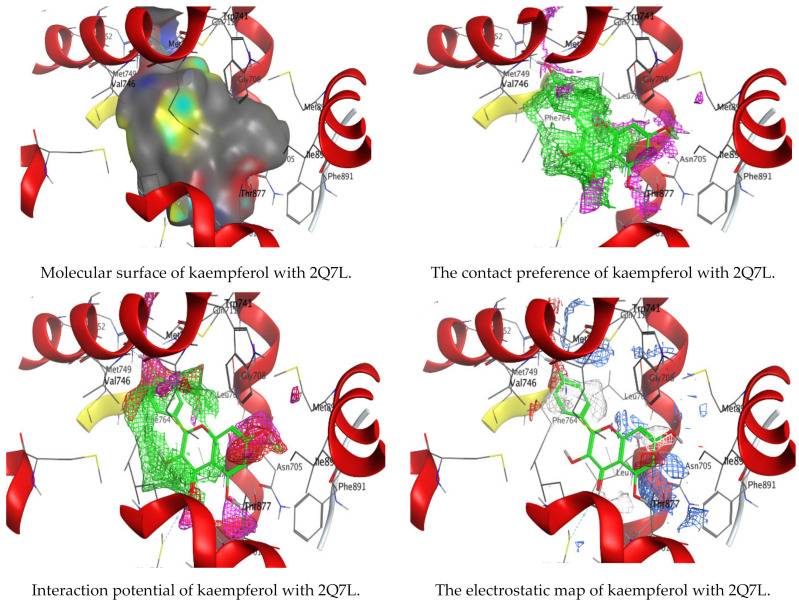
Molecular docking process of kaempferol with 2Q7L protein.

**Figure 9:**
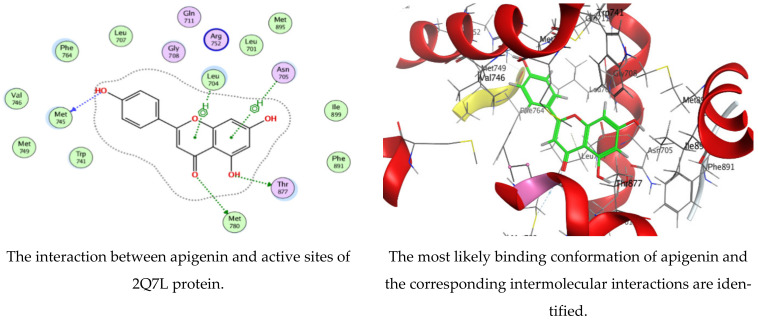

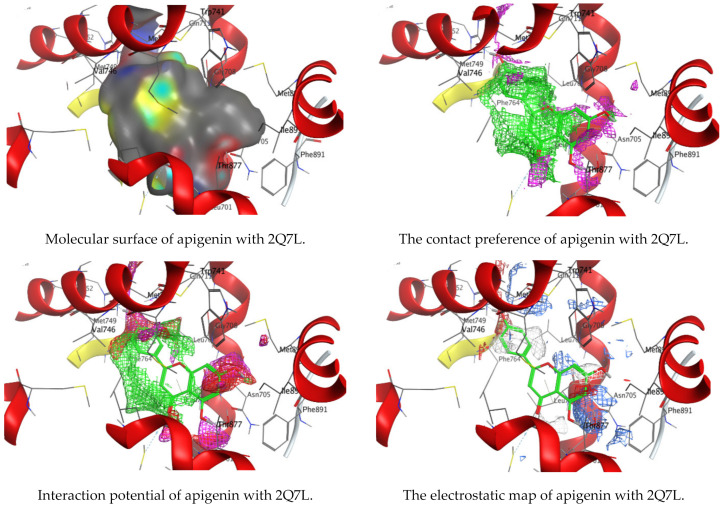
Molecular docking process of apigenin with 2Q7L protein.

The MOE-Dock program’s default parameters were employed for the molecular docking of the kaempferol and apigenin. The ligands were permitted to be flexible to determine the proper conformations of the ligands and to produce the least energy structures. The best ligand conformations were examined with Prostate Cancer (2Q7L) for their binding interaction. From the docking studies, it was observed that both ligands (kaempferol and apigenin) are good inhibitors of the 2Q7L enzyme, with low scores for binding free energy of −6.61458 kcal/mol and −6.47986 kcal/mol, respectively. The binding mode of the inhibitors kaempferol and apigenin are within the active site of 2Q7L (Figure 8 and Figure 9). Kaempferol was bound deeply to the binding cavity of Prostate Cancer (2Q7L), interacting with the residues MET 745, MET 780, THR 877, LEU 704, ASN 705, and PHE 764 via the O 23, O 25, O 26, and three 6-rings.

Likewise, apigenin showed similar interactions with the binding site residues MET 745, MET 780, THR 877, LEU 704, and ASN 705 via the O 24, O 2, O 27, and two 6-rings. The results are summarized in Table 5 and Table 6. In a recent investigation, the anticancer activity of chlorogenic acid, rutin, chrysoeriol, and kaempferol against breast cancer and PC3 cells, respectively, was documented via molecular docking protocol [51,52]. Another application of molecular docking was performed on the activity of the natural molecules of *aloe vera* gel doped with chitosan nanoparticles binding to *Helicobacter pylori* proteins [17]. Experimental findings by Qanash et al. [3] regarding the molecular docking of natural compounds such as chlorogenic acid confirmed its bacteriostatic action against *Proteus vulgaris* and antivirus activities against human corona virus (HCoV-229E). Moreover, natural exudates from some fungi were docked with *Bacillus subtilis* and *Candida albicans* [53] to elucidate its antimicrobial potential.

## 4. Conclusions

The phytochemical characterization of *Artemisia judaica* extract via HPLC showed several compounds with different concentrations belonging to phenolics and flavonoids. The antimicrobial activity of *A. judaica* extract was documented against *B. subtilis*, *E. coli*, *S. aureus*, *K. pneumonia*, and *C. albicans*, but not towards tested filamentous fungus (*A. niger*). In addition, the potent anticancer activity of the *A. judaica* extract against PC3 and the limited toxicity of normal Vero cells were revealed. Notably, the biological activities were found to be significantly enhanced when the extract was loaded with CNPs. Furthermore, the docking experiments showed an estimate of the ligand’s inhibitory actions. These demonstrated that the inhibitors kaempferol and apigenin were well-suited to the active site of the androgen receptor Prostate Cancer (2Q7L) and interacted with the residues essential for their biological activities. In short, *A. judaica* is a rich source for compounds with potential therapeutic properties, including antimicrobial and anticancer activities. The low toxicity of the extract towards Vero cells should highlight the promising future for the active compound(s) within the extract to be used in human medicine.

## Figures and Tables

**Table 1:** Polyphenolic and flavonoid compounds detected in *Artemisia judaica* extract.

Compound	Retention Time	Area	Area (%)	Concentration (µg/mL)
Gallic acid	3.46	75.38	1.41	225.96
Chlorogenic acid	4.21	191.54	3.60	1089.58
Catechin	4.79	16.97	0.32	167.62
Methyl gallate	5.66	12.29	0.23	27.68
Caffeic acid	6.06	44.82	0.84	134.14
Syringic acid	6.54	80.26	1.51	244.74
Rutin	8.25	11.77	0.22	55.64
Ellagic acid	8.44	12.86	0.24	134.23
Coumaric acid	9.25	5.31	0.10	5.46
Vanillin	9.58	569.10	10.68	691.55
Ferulic acid	10.28	66.65	1.25	139.74
Naringenin	10.53	53.46	1.00	202.14
Daidzein	12.34	28.78	0.54	69.63
Quercetin	12.85	147.66	2.77	714.97
Cinnamic acid	13.59	109.08	2.05	83.29
Apigenin	14.53	1629.47	30.58	3794.32
Kaempferol	15.01	652.66	12.25	3916.34
Hesperetin	15.61	11.53	0.22	25.09
Unknown	2.64	1057.93	19.86	Undetected
Unknown	3.00	104.30	1.96	Undetected
Unknown	10.06	327.02	6.14	Undetected
Unknown	12.64	119.03	2.23	Undetected

**Table 2:** Minimum inhibitory concentration (MIC) of *Artemisia judaica* extract and chitosan nanoparticles loaded extract (CNPsLE) towards multiple pathogenic bacterial and fungal species.

Microorganisms	MIC µg/mL	
	CNPsLE	Extract
*Bacillus subtilis*	0.97	62.5
*Staphylococcus aureus*	3.90	15.65
*Escherichia coli*	1.95	15.62
*Klebsiella pneumoniae*	4.10	31.25
*Candida albicans*	15.62	31.25
*Aspergillus niger*	-	-

**Table 3:** Cytotoxicity of different concentrations of *Artemisia judaica* extract and chitosan nanoparticles loaded extract against normal Vero cells.

Concentration µg/mL	Extract			Chitosan Nanoparticles Loaded Extract		
	Mean OD	Viability %	Toxicity %	Mean OD	Viability %	Toxicity %
0	0.786 ± 0.006	100.0	0.00	0.786 ± 0.006	100.0	0.00
31.25	0.784 ± 0.003	99.75	0.25	0.738 ± 0.003	93.89	6.11
62.5	0.777 ± 0.009	98.85	1.15	0.358 ± 0.004	45.63	54.37
125	0.558 ± 0.005	71.03	28.97	0.019 ± 0.032	13.10	86.90
250	0.131 ± 0.005	16.80	83.29	0.774 ± 0.788	7.55	92.45
500	0.019 ± 0.000	2.42	97.58	0.031 ± 0.002	3.94	96.06
1000	0.018 ± 0.001	2.25	97.75	0.024 ± 0.003	3.10	96.90
IC_50_ ± SD	173.74 ± 2.13 µg/mL			73.89 ± 0.8 µg/mL		

OD; optical density.

**Table 4:** Cytotoxicity of different concentrations of *Artemisia judaica* extract and chitosan nanoparticles loaded extract (CNPsLE) against the PC3 cell line.

Concentration µg/mL	Extract			Chitosan Nanoparticles Loaded Extract		
	Mean OD	Viability %	Toxicity %	Mean OD	Viability %	Toxicity %
0	0.856 ± 0.003	100.0	0.00	0.856 ± 0.003	100.0	0.00
31.25	0.729 ± 0.005	85.20	14.80	0.224 ± 0.004	26.21	73.80
62.5	0.428 ± 0.001	50.04	49.96	0.118 ± 0.006	13.79	86.21
125	0.182 ± 0.008	21.30	78.70	0.083 ± 0.008	9.74	90.26
250	0.058 ± 0.003	6.78	93.22	0.052 ± 0.003	6.11	93.89
500	0.020 ± 0.001	2.30	97.70	0.029 ± 0.001	3.35	96.65
1000	0.018 ± 0.001	2.10	97.90	0.025 ± 0.001	2.41	97.59
IC_50_± SD	76.09 ± 0.6 µg/mL			20.8 ± 0.23 µg/mL		

OD; optical density.

**Table 5:** Docking scores and energies of kaempferol and apigenin with prostate cancer (2Q7L) receptors.

Mol	rseq	mseq	S	rmsd_refine	E_conf	E_place	E_score1	E_refine	E_score2
Kaempferol	1	1	−6.61458	1.230687	4.854888	−82.8657	−12.7112	−32.7628	−6.61458
Kaempferol	1	1	−6.54383	1.119653	6.537412	−92.123	−12.5656	−28.8529	−6.54383
Kaempferol	1	1	−6.54332	1.526945	12.30441	−89.1824	−12.9152	−27.8703	−6.54332
Kaempferol	1	1	−6.4475	1.046071	6.208238	−85.183	−13.053	−30.3745	−6.4475
Kaempferol	1	1	−6.37303	0.916227	7.082326	−80.5611	−12.9719	−31.4932	−6.37303
Apigenin	1	2	−6.47986	0.861858	−26.2797	−84.5134	−12.2088	−32.4797	−6.47986
Apigenin	1	2	−6.47244	2.015834	−29.3779	−72.5522	−12.15	−27.6243	−6.47244
Apigenin	1	2	−6.27735	0.797183	−25.3818	−88.9263	−13.4198	−30.0022	−6.27735
Apigenin	1	2	−6.17308	1.56109	−25.6403	−63.8105	−12.9929	−29.4521	−6.17308
Apigenin	1	2	−6.16644	0.588309	−27.4292	−88.1041	−12.1956	−29.6247	−6.16644

**Table 6:** Interaction of kaempferol and apigenin with Prostate Cancer (2Q7L) receptors.

Mol	Ligand		Receptor				Interaction	Distance	E (kcal/mol)
Kaempferol	O	23	O	MET	745	(A)	H-donor	2.90	−1.5
	O	25	SD	MET	780	(A)	H-donor	3.78	−0.9
	O	26	OG1	THR	877	(A)	H-donor	3.00	−0.5
	6-ring		CB	LEU	704	(A)	pi-H	4.33	−0.5
	6-ring		CA	ASN	705	(A)	pi-H	4.47	−0.5
	6-ring		CD1	PHE	764	(A)	pi-H	3.88	−0.5
Apigenin	O	24	O	MET	745	(A)	H-donor	2.89	−1.6
	O	26	SD	MET	780	(A)	H-donor	3.78	−1.1
	O	27	OG1	THR	877	(A)	H-donor	3.07	−0.5
	6-ring		CB	LEU	704	(A)	pi-H	4.36	−0.5
	6-ring		CA	ASN	705	(A)	pi-H	4.49	−0.5

## Data Availability

All data are available within the manuscript.
